# Bilateral nipple-sparing mastectomy and breast reconstruction in BRCA1 mutation-positive simultaneous bilateral breast cancer: A case study

**DOI:** 10.1016/j.ijscr.2021.105788

**Published:** 2021-03-17

**Authors:** Kimiyasu Yoneyama, Motohito Nakagawa, Asuka Hara

**Affiliations:** Department of Breast Surgery, Hiratsuka City Hospital, 1-19-1 Minamihara, Hiratsuka-shi, Kanagawa 254-0065, Japan

**Keywords:** BRRM, bilateral risk-reducing mastectomy, Bt, total mastectomy, CRRM, contralateral risk-reducing mastectomy, IBTR, ipsilateral breast tumor recurrence, NSM, nipple-sparing mastectomy, SSM, skin-sparing mastectomy, Bilateral breast cancer, BRCA mutation, Breast reconstruction

## Abstract

**Background:**

Mutation-positive patients who develop unilateral breast cancer require different treatments, such as prophylactic mastectomy of the contralateral breast, from those used for other breast cancer patients. If a mutation is found before surgery, it is necessary to consider a surgical procedure that includes reconstruction. For BRCA mutation-positive patients, a suitable treatment must be selected. In Japan, a test for BRCA mutation has been covered by health insurance since 2020, making it possible to preoperatively test patients who are suspected of being positive. We report a case of simultaneous bilateral breast cancer that was found to be BRCA mutation-positive preoperatively and underwent bilateral subcutaneous mastectomy and breast reconstruction.

**Case presentation:**

A 57-year-old woman was admitted to our hospital after a breast cancer screening revealed a mass in the left breast. She had a family history of breast cancer, including her sister, aunt, and cousin. She was suspected of being malignant with a mass on both sides of her breast on imaging. She underwent needle biopsy and was diagnosed as having bilateral invasive ductal carcinoma, for which she was placed on preoperative chemotherapy. Due to the strong family history of bilateral breast cancer, the patient was recommended to undergo a BRCA gene-mutation test and she consented. The result was positive for BRCA1 mutation. Although it was judged that bilateral breast-conserving surgery was sufficiently possible, bilateral subcutaneous mastectomy and breast reconstruction were performed based on BRCA mutation-positive status.

**Discussion:**

Performing a preoperative BRCA test may change the surgical procedure.

BRCA tests are beneficial to patients, but the timing of the tests is important. Care must be taken not to force the patient.

**Conclusions:**

Knowing whether the patient is BRCA mutation-positive is extremely important for selecting surgical procedures and treatment methods. BRCA testing should be recommended for patients who are strongly suspected of being positive, but the decision should be the patient’s. It is therefore necessary to provide accurate information and engage in a dialogue with the patient, but the medical staff should not pressure the patient to have the test.

## Highlights

• BRCA-positive breast cancer with bilateral subcutaneous mastectomy and breast reconstruction.
• The patient was able to be diagnosed preoperatively.
• Performing a preoperative BRCA test may change the surgical procedure.
• Increasing numbers of BRCS tests may change surgical treatment strategies.
• This is the first report in Japan.

## 1 Background

Breast cancer is the most common cancer in Japan, affecting 40,000 people annually and killing 10,000. Breast cancer that develops in both breasts is called bilateral breast cancer, and simultaneous bilateral breast cancer accounts for 6.0% of such cases, according to the 2015 Annual Breast Cancer Registry (final edition) of the Japanese Breast Cancer Society [1]. Patients with bilateral breast cancer may have some genetic predisposition. One such predisposing factor is a mutation of the BRCA gene. BRCA mutations are found in 5%–10% of breast cancer patients. Until recently, BRCA genetic testing was not covered by medical insurance in Japan and could not be performed before surgery. However, it began to be covered by health insurance in Japan in April 2020. For patients in whom breast cancer has already developed, the test is indicated for cases such as the onset of two or more primary breast cancers and the presence of breast or ovarian cancer among third-degree relatives. It is critical to determine whether the patient has the mutation before selecting the method for treatment, including surgery. We report a case of simultaneous bilateral breast cancer that was found to be BRCA mutation-positive preoperatively and in which the patient underwent bilateral total subcutaneous mastectomy and breast reconstruction.

This work was written in accordance with the SCARE criteria [2].

## 2 Case presentation

**Fig. 1:**
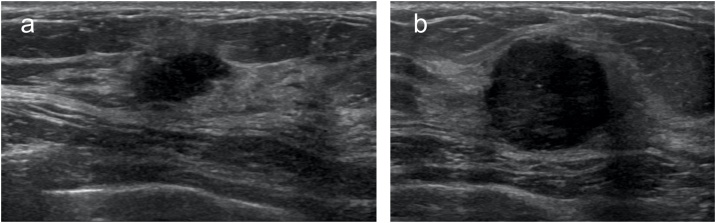
Breast ultrasound images at the first visit. a) The tumor on the right is a hypoechoic mass with unclear boundaries, irregular margins, and non-uniform internal echo. b) The tumor on the left is a well-defined, hypoechoic mass with irregular margins and relatively uniform internal echo.

**Fig. 2:**
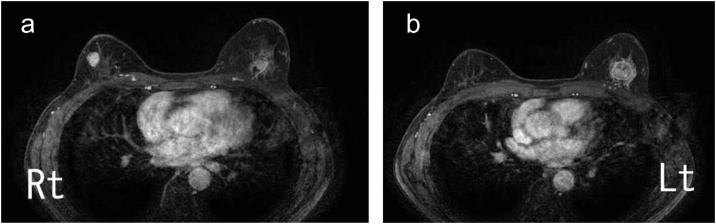
Contrast-enhanced breast magnetic resonance images at the first visit. a) In the right breast, an 18-mm, well-defined, marginal spicula-like lobulated tumor that is strongly enhanced from an early stage is observed. b) In the left breast, a 21-mm, well-defined circular tumor with irregular margins that is strongly enhanced from an early stage is observed.

A 57-year-old woman was admitted to our hospital after a breast cancer screening revealed a mass in the left breast. She had a family history of breast cancer, including her sister, aunt, and cousin. A mass 4 cm in diameter was palpable on the outside of her left breast and another mass 2 cm in diameter was palpable on the outside of her right breast. Mammography showed no clear tumor on the right, but a well-defined tumor on the left was judged to be category 4. Ultrasonography revealed hypoechoic masses with irregular shapes, unclear boundaries, and irregular margins on both breasts (Fig. 1). Magnetic resonance images also showed a well-defined, irregularly marginal mass with deep staining in both breasts at an early stage (Fig. 2). She underwent needle biopsy and was diagnosed as having bilateral invasive ductal carcinoma.

Immunohistochemical examination revealed that the left tumor was ER-positive, PgR-negative, HER2-negative, and Ki-67 90%; the right tumor was ER-positive, PgR-positive, HER2-negative, and Ki-67 25%. Ki-67 expression was high on both sides, and the patient wished to preserve the breast, so preoperative chemotherapy was performed. For chemotherapy, epirubicin (100 mg / m^2^) and cyclophosphamide (600 mg / m^2^) were administered 4 times every 3 weeks, and docetaxel (75 mg / m^2^) was administered 4 times every 3 weeks. Because of the strong family history of bilateral breast cancer, the patient was recommended to undergo a BRCA gene-mutation test and she consented. The result was positive for BRCA1 mutation. Patients performed only BRCA tests, not other genetic tests.

**Fig. 3:**
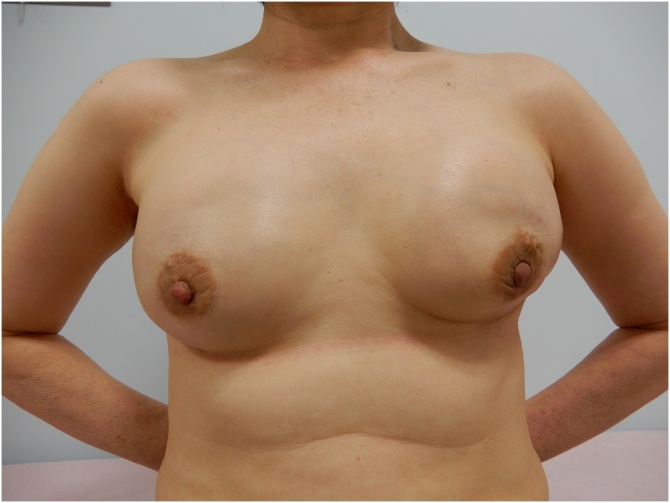
Three months after surgery. The positions of the left and right nipples are the same height, and the results of plastic surgery are good.

Imaging at the end of preoperative chemotherapy showed that both breast masses had shrunk, and it was judged that breast-conserving surgery was sufficiently possible. However, considering the BRCA mutation-positive status, we decided to reconsider the surgical procedure and chose instead to perform bilateral subcutaneous mastectomy and breast reconstruction. The mastectomy was performed by a breast surgeon with 30 years of experience, and the reconstruction was performed by a plastic surgeon with 25 years of experience. Both sides of the sentinel lymph node were negative for metastasis. The postoperative course was good and the plastic surgery was also good (Fig. 3). Postoperative histopathological examination revealed a tumor with a diameter of 5 mm in the right breast and pathological complete response in the left breast without any tumor. The patient was started on an aromatase inhibitor and is currently being followed up. There is no recurrence 5 months after the operation.

Written informed consent was obtained from the patient for publication of this case report and accompanying images.

## 3 Discussion

Bilateral breast cancer is breast cancer that develops in both breasts, but the frequency of simultaneous bilateral breast cancer might depend on the extent of preoperative examination. Contralateral lesions are often found by various diagnostic imaging, especially breast magnetic resonance imaging. Patients with bilateral breast cancer, whether simultaneous or metachronous, may have some predisposing factors.

BRCA mutations are found in 5%–10% of breast cancer patients. Mutation-positive patients who develop unilateral breast cancer require different treatments, such as prophylactic mastectomy of the contralateral breast, from those used for other breast cancer patients. If a mutation is found before surgery, it is necessary to consider a surgical procedure that includes reconstruction.

BRCA genetic testing began to be covered by health insurance in Japan in April 2020. Previously, BRCA gene mutations were not detected, and therefore the same measures as in sporadic breast cancer were taken.

For those who have already developed breast cancer, insurance covers women aged 45 years or younger, women under the age of 60 years with triple-negative breast cancer, women with two or more primary breast cancers, women whose third-degree relatives have had breast or ovarian cancer, and men with breast cancer. Such cases are considered highly likely to have the gene mutation, and thus BRCA genetic testing is recommended.

For BRCA mutation-positive patients, the treatment is generally one of the following: partial mastectomy, full mastectomy, mastectomy and breast reconstruction, or subcutaneous mastectomy and breast reconstruction. Which of these is best for the patient must be thoroughly considered.

Intramammary recurrence rate after breast-conserving surgery in BRCA mutations

Breast-conserving surgery for premenopausal breast cancer cases with BRCA mutation is relatively contraindicated in the US National Comprehensive Cancer Network guidelines [3]. The Japanese Breast Cancer Society clinical practice guidelines recommend that mastectomy be performed for patients with BRCA mutations, even if breast-conserving surgery is possible, unless the patient strongly desires breast-conserving surgery [4]. The rationale for this is that ipsilateral breast tumor recurrence (IBTR) after breast-conserving surgery is likely to be high in the BRCA-mutation group. IBTR is reported to be 20%–40% over a 6- to 10-year observation period [[5], [6], [7], [8])]. In these case-control studies, there was no significant difference in the IBTR rate between mutation-positive patients and the control group, but the recurrence rate varied depending on the observation period. Reviewing studies with an observation period of 10 years or less, Robson et al. reported that IBTR was 12% in the mutation group and 8% in the control group (p = 0.68) [5], and Pierce et al. reported that the mutation group was 12% and control group 9% (p = 0.19) [8], showing no significant difference between studies with a relatively short observation period. According to a report by Haffty et al. with an observation period of 12 years, the IBTR rate was significantly different between the mutation group (49%) and the control group (21%) (p = 0.007), and late recurrence was particularly common [9]. In a meta-analysis of 10 studies, there was no significant difference in the IBTR rate with or without BRCA mutations during the observation period of less than 7 years (mutation group 11.7% vs. non-mutation group 8.9%, p = 0.51). It has been reported that the IBTR rate increases over a long period of time (mutation group 23.7% vs. non-mutation group 15.9%, p = 0.003) [10]. This is considered to be due to an increase in the incidence of new breast cancer, and it is often reported that the site of recurrence is in an area distant from the initial site. As for other factors related to IBTR, there was no difference in the IBTR rate between the BRCA1 and BRCA2 mutation groups, and chemotherapy and oophorectomy were associated with reduced risk of IBTR [10].

### 3.1 Prognosis after breast-conserving surgery in BRCA mutations

According to a meta-analysis of 10 studies, the prognosis of the BRCA-mutation group undergoing breast-conserving surgery was not different from that of the non-mutation group [9]. According to a report by Pierce et al., the 10-year survival rate in the BRCA-mutation group was 92.1% in the breast-conserving surgery sub-group and 91.8% in the total breast resection sub-group, showing no difference in prognosis due to differences in surgical procedures [11].

### 3.2 SSM or NSM

Prophylactic mastectomy may be performed in patients positive for BRCA mutations. Prophylactic mastectomy includes bilateral risk-reducing mastectomy (BRRM) and contralateral risk-reducing mastectomy (CRRM). Both BRRM and CRRM reduce the risk of breast cancer by 90% or more, according to multiple reports [[12], [13], [14])]. However, even after RRM, the risk of developing breast cancer remains, albeit small, owing to the presence of the residual mammary gland tissue [15,16]. RRM as a surgical procedure can also be applied to breast cancer patients, and RRM procedures include total mastectomy (Bt), skin-sparing mastectomy (SSM), and nipple-sparing mastectomy (NSM). For RRM, either SSM or NSM with breast reconstruction is often selected from the viewpoint of maintaining plastic surgery. In Europe and the United States in particular, the proportion of NSM is high, and it is reported that the satisfaction level is much higher than that of Bt and SSM [17]. However, there is a concern that the risk of developing breast cancer afterwards may increase given that NSM often leaves a larger amount of mammary gland tissue compared with other surgical methods [15,18]. However, investigations into the subsequent risk of developing breast cancer, even with NSM for BRCA mutation carriers, revealed no significant difference between SSM and Bt [17,19]. When considering surgery similar to RRM for breast cancer patients, it is important to balance risk reduction and plastic surgery. Although it is impossible to completely eliminate the risk, if risk reduction is pursued, excision that eliminates as much residual breast tissue as possible is necessary. However, if plastic surgery is pursued, some residual mammary gland tissue is inevitable, such as flap preparation on the premise of breast reconstruction, consideration of the peripheral mastectomy range, and preservation of the nipple areola. It is difficult to achieve these two goals of risk reduction and also carry out plastic surgery at the same time, but it is necessary to consider the patient's wishes and thoroughly discuss with each patient which and how much emphasis should be placed on the surgery.

### 3.3 Timing of genetic testing

For breast cancer surgery cases, it is necessary to provide genetic counseling before surgery, depending on the situation, for cases with a high possibility of BRCA mutation based on considerations such as family history, age of onset, and breast cancer subtype. It is necessary to discuss whether to undergo a genetic test and what to do if the patient is mutation-positive. In particular, if breast-conserving surgery is planned, there are many cases in which total mastectomy may be selected according to the results of genetic testing. Based on the psychological state of the patient, it is important to perform genetic testing so that the patient will not have regrets later. It is probable that even in our case, bilateral partial mastectomy would have been selected. However, it is also necessary to consider that the patient must consider, accept, and make decisions based on factors other than genetic mutations. If the likelihood of mutation is not high, it is important to provide treatment in the same manner as in sporadic breast cancer and consider genetic counseling at a time appropriate for the patient after treatment. The decision to undergo a genetic test should be the patient’s. Genetic counseling should provide accurate information and dialogue for self-determination, and refrain from strongly pressuring the patient to have the test.

## 4 Conclusions

We encountered a case of simultaneous bilateral breast cancer that was found to be BRCA mutation-positive preoperatively and underwent bilateral total mastectomy and breast reconstruction. Knowing whether the patient is BRCA mutation-positive patients is extremely important for selecting surgical procedures and treatment methods. There are advantages and disadvantages to knowing about gene mutations, including those in the patient’s family, but there are many potential benefits for patients. BRCA genetic testing should be recommended for patients who are strongly suspected of being BRCA-positive. However, the decision to undergo a genetic test should be the patient’s. It is therefore necessary to provide accurate information and engage in a dialogue with the patient, but the medical staff should not pressure the patient to have the test.

## Declaration of Competing Interest

The authors declare that there is no conflict of interest regarding the publication of this article.

## Sources of funding

Our study has not received any grant of funding.

## Ethical approval

Our institution does not require ethical approval for case reports that are deidentified and collected retrospectively.

## Consent

Written informed consent was obtained from the patient for publication of this case report and accompanying images.

## Author contribution

**Kimiyasu Yoneyama**: Conceptualization, Investigation, Resources, Writing – Original draft preparation, Writing – Review and editing.

**Asuka Hara**: Conceptualization, Investigation.

**Motohito Nakagawa**: Administration, Review.

## Registration of research studies

Not applicable.

## Guarantor

Kimiyasu Yoneyama.

## Provenance and peer review

Not commissioned, externally peer-reviewed.
